# DNAJC17 is localized in nuclear speckles and interacts with splicing machinery components

**DOI:** 10.1038/s41598-018-26093-1

**Published:** 2018-05-17

**Authors:** A. Pascarella, G. Ferrandino, S. C. Credendino, C. Moccia, F. D’Angelo, B. Miranda, C. D’Ambrosio, P. Bielli, O. Spadaro, M. Ceccarelli, A. Scaloni, C. Sette, M. De Felice, G. De Vita, E. Amendola

**Affiliations:** 1 Dipartimento di Medicina Molecolare e Biotecnologie Mediche, Università degli Studi di Napoli Federico II, Napoli, Italy; 2 Istituto di Ricerche Genetiche G. Salvatore, Biogem s.c.ar.l, Ariano Irpino (AV), Italy; 3 Proteomics & Mass Spectrometry Laboratory, ISPAAM, National Research Council, Napoli, Italy; 4 Laboratory of Neuroembryology, Fondazione Santa Lucia, 00143 Rome, Italy; 5 Department of Biomedicine and Prevention, Università di Roma Tor Vergata, 00133 Rome, Italy; 6 Institute of Human Anatomy and Cell Biology, Università Cattolica del Sacro Cuore, 00168 Rome, Italy

## Abstract

DNAJC17 is a heat shock protein (HSP40) family member, identified in mouse as susceptibility gene for congenital hypothyroidism. DNAJC17 knockout mouse embryos die prior to implantation. In humans, germline homozygous mutations in DNAJC17 have been found in syndromic retinal dystrophy patients, while heterozygous mutations represent candidate pathogenic events for myeloproliferative disorders. Despite widespread expression and involvement in human diseases, DNAJC17 function is still poorly understood. Herein, we have investigated its function through high-throughput transcriptomic and proteomic approaches. DNAJC17-depleted cells transcriptome highlighted genes involved in general functional categories, mainly related to gene expression. Conversely, DNAJC17 interactome can be classified in very specific functional networks, with the most enriched one including proteins involved in splicing. Furthermore, several splicing-related interactors, were independently validated by co-immunoprecipitation and *in vivo* co-localization. Accordingly, co-localization of DNAJC17 with SC35, a marker of nuclear speckles, further supported its interaction with spliceosomal components. Lastly, DNAJC17 up-regulation enhanced splicing efficiency of minigene reporter in live cells, while its knockdown induced perturbations of splicing efficiency at whole genome level, as demonstrated by specific analysis of RNAseq data. In conclusion, our study strongly suggests a role of DNAJC17 in splicing-related processes and provides support to its recognized essential function in early development.

## Introduction

DnaJ proteins, also known as Hsp40, are a set of highly conserved proteins typically acting as co-factors of Hsp70 molecular chaperones. They drive functional specificity to the otherwise ubiquitous Hsp70 partners, either by targeting them at precise cell compartments, or by delivering specific clients to Hsp70^1^. DNAJ proteins, although very different from each other, share the presence of the J domain, whose main function is the stimulation of Hsp70 ATPase activity. Based on their domain architecture, DNAJ are classified in three different groups (A, B and C). Dnajc17 belongs to group C, bearing the J domain at its N-terminal region and a RNA recognition motif (RRM) located at the protein C-terminus^1^, (UniProt: www.uniprot.org). It was identified as modifier gene for congenital hypothyroidism (CH) with thyroid dysgenesis (TD) by genetic linkage analysis of a polygenic mouse model of CH with TD^2,3^. Importantly, *Dnajc17* null mouse embryos die between morula and blastocyst stages, thus demonstrating that this gene plays an essential role since the very early stages of development^3^.

Mutations in Dnajc17 have been associated with several human disorders. Patel and collaborators identified homozygous truncating mutation that segregated in a family with an apparently novel syndrome of retinitis pigmentosa and hypogammaglobulinemia, thus highlighting Dnajc17 as a candidate gene for retinal dystrophy^4^. Two different missense mutations in exon 11 of the human *DnaJc17* gene were identified in patients with essential thrombocythaemia by two independent studies^5,6^. Furthermore, *DnaJc17* transcripts were found to be subjected to differential alternative splicing in blood cells from Autism Spectrum Disorder (ASD)^7^. Thus, although the specific function(s) of DNAJC17 is still unknown, it is likely that corresponding gene alterations result in detrimental effects.

*Dnajc17* is the vertebrate ortholog of Saccharomyces Cerevisiae Cwc23, an essential protein involved in pre-mRNA splicing^8,9^. However, there is no functional data about the implication of DNAJC17 in this process. Notably, DNAJC17 contains a RRM motif, which represents the most common RNA binding motif in vertebrates and characterizes many splicing factors, like serine-arginine rich (SR) proteins and heterogeneous ribonucleoproteins (hnRNPs)^10^. In this study, we have investigated the functional role of human DNAJC17 by combined transcriptomic/proteomic approaches. Additional protein localization experiments provided information on the predominant nuclear localization of the protein, where it co-localizes with splicing speckles. Minigene splicing assays, together with a splicing-specific analysis of RNAseq data, suggested DNAJC17 direct role in cell mechanisms related to pre-mRNA splicing. By introducing novel functional informations, this work complements preliminary data on its ortholog in yeast^9^, which were obtained with gene deletion mutants, and confirmed the involvement of molecular chaperones in the splicing machinery at splicing speckles.

## Results

### Gene expression profiling highlights general biological processes influenced by DNAJC17 expression

**Figure 1:**
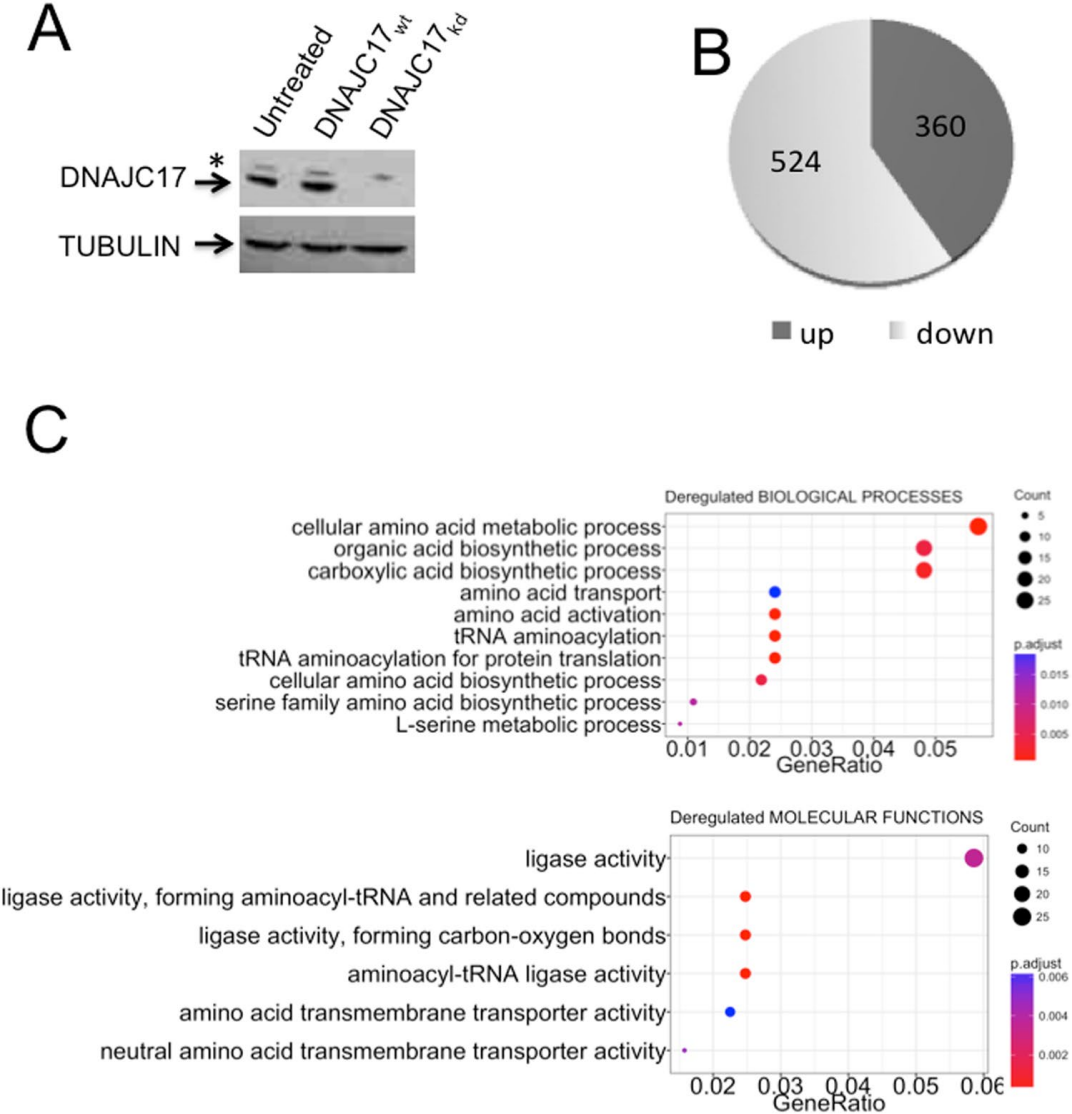
Gene expression profile analysis of DNAJC17-depleted cells. (**A**) HeLa cells were transfected with either siRNA specific for DNAJC17 (DNAJC17_kd_) or scramble (DNAJC17_wt_). Western blot analysis at 96 h after transfection revealed the specific decline of DNAJC17 (arrow) only in DNAJC17_kd_ cells. Asterisk indicates the aspecific band recognized by the antibody. Tubulin was used as normalization. (**B**) DNAJC17_kd_ transcriptome was compared with that of DNAJC17_wt_ cells. Pie chart shows that, among the 884 regulated genes, 524 were down-regulated and 360 were up-regulated. **(C)** Gene Ontology functional analysis showing Biological Process (upper) and Molecular Function (lower) terms significantly enriched in deregulated genes. Enriched terms are represented in dotplot by *Count* (the number of deregulated genes annotated with the term), *p-adjust* (corrected *p*-value of hypergeometric test) and *GeneRatio* (the ratio between *Count* and the number of all genes annotated with the term).

To shed light on DNAJC17 function, we analyzed gene expression profiles induced by its depletion. HeLa cells were transfected with either a DNAJC17-specific siRNA (DNAJC17_kd_) or a scramble siRNA, as a control (DNAJC17_wt_). Western blot analysis performed at 96 h after siRNA transfection confirmed a strong reduction of DNAJC17 levels in DNAJC17_kd_ cells as compared to control DNAJC17_wt_ cells (Fig. 1A). DNAJC17 silencing effect was then investigated by comparing DNAJC17_kd_ cells transcriptome with that of DNAJC17_wt_ cells through high-throughput sequencing of total RNA (RNA-Seq). Three replicates for both conditions were sequenced producing about 7 × 10^7^ reads for sample, that were mapped to reference genome (Homo sapiens, GRCh37) using TopHat aligner^11^. Correctly mapped reads were then processed by Cufflinks^12^ to assemble genes and transcripts and to calculate their relative expression levels. A total of 20825 assembled genes were selected for further analysis to highlight differential gene expression. Using absolute fold change ≥1.5 with corrected p-value ≤ 0.05 as cut-off, we identified 884 genes (4,24% of expressed genes) as differentially expressed in DNAJC17_kd_ cells (Table S1). Among them, 360 (40%) were up-regulated and 524 (60%) down-regulated in response to DNAJC17 depletion (Fig. 1B and Table S1). Gene Ontology (GO) terms enrichment analysis using R package clusterProfiler^13^ revealed that differentially regulated genes are enriched in functional categories representing ten Biological Processes (q-value ≤ 0.05) and seven Molecular Functions (q-value ≤ 0.05) (Fig. 1C). In both groups, enriched terms are related to protein synthesis-related processes, mainly regarding amino acid metabolism and aminoacyil-tRNA synthesis. These results suggest a pleiotropic effect of DNAJC17 on several general cellular functions, which may underlie its essential role during early embryogenesis^3^.

### Proteomic analysis of DNAJC17 interactome

**Figure 2:**
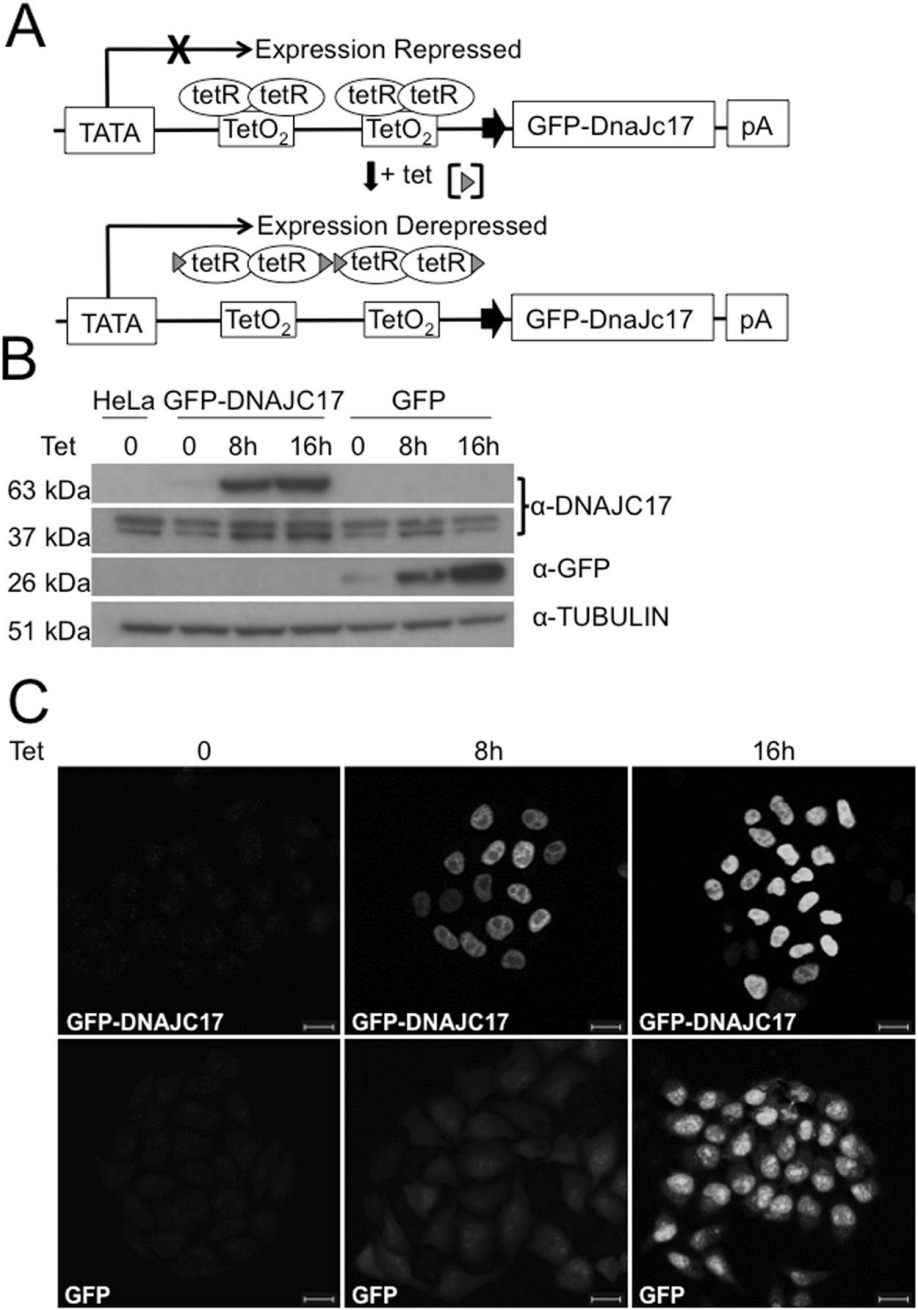
Schematic representation of the Tet-inducible GFP-DNAJC17 fusion protein. (**A**) The binding of tetracycline to the Tet repressor activates the promoter controlling GFP-DNAJC17 expression. (**B**) Western blot analysis of whole HeLa (lane 1), GFP-DNAJC17 (lanes 2–4) and GFP (lanes 5–7) cells extracts, showing the expression of GFP-DNAJC17 in a tetracycline dose-dependent manner. GFP-DNAJC17 (upper panel) and endogenous DNAJC17 (second panel) have been revealed by the same western blot with DNAJC17 antibody, captured at lower or higher exposure time, respectively. Molecular mass of each detected protein is indicated on the left. (**C**) Immunofluorescence at the same time points as (**B**) shows that GFP-DNAJC17 localizes in the nucleus, while GFP alone localize also in the cytoplasm.

As DNAJ proteins generally function as co-factors in larger protein complexes^14^, we further characterized DNAJC17 function using a proteomic approach to analyze its protein interactome upon expression of a GFP-tagged protein. To generate a GFP-DNAJC17-expressing cell line, the DNAJC17 cDNA from Sv129 mouse strain was cloned in Flp-In T-REx expression vector in frame with GFP (Fig. 2A). Such expression vector was transfected into HeLa cells to obtain a cell line exhibiting tetracycline-inducible expression of GFP-DNAJC17 (GFP-DNAJC17 cells). As a control, we also generated stable HeLa cell line with tetracycline (Tet)-inducible expression of GFP (GFP cells). After transfection of each vector, HeLa cells were antibiotic-selected and the pools of colonies obtained were used for further analyses.

Western blot analysis of whole protein extract from control HeLa cells, GFP-DNAJC17 and GFP cells confirmed the expression of either GFP-DNAJC17 or GFP in a tetracycline-dependent manner at both 8 and 16 h of treatment (Fig. 2B). Immunofluorescence analysis at the same time points showed that GFP-DNAJC17 exhibits nuclear localization, similarly to the endogenous DNAJC17 in thyroid cells^3^, whereas GFP alone is detectable both in the nucleus and in the cytoplasm (Fig. 2C). For all further experiments, we used 16 h of Tet treatment.

**Figure 3:**
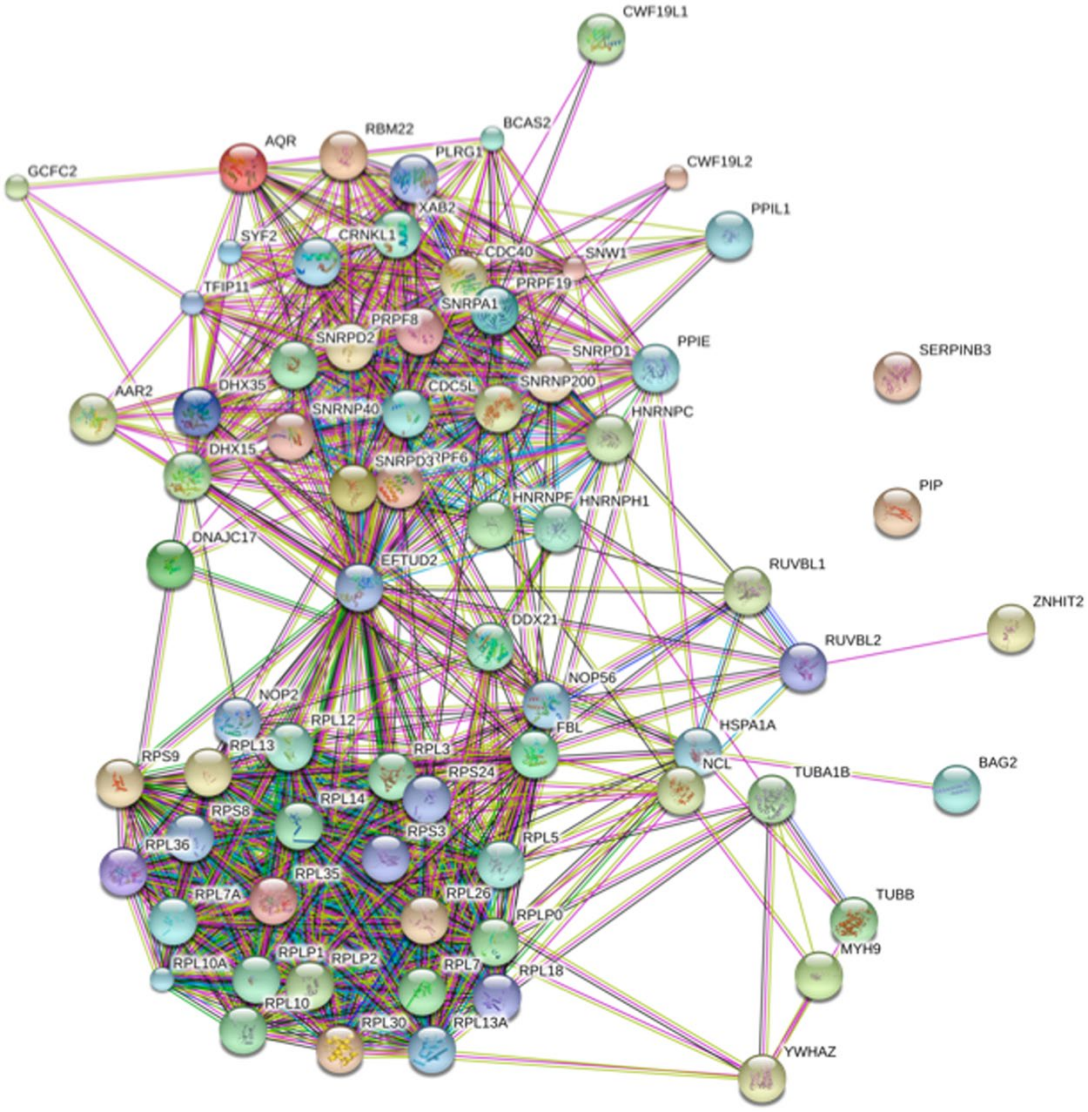
Map of the Interactome Network showing 70 proteins interacting with DNAJC17 protein. In particular, the reported interactors of the GFP-DNAJC17 fusion protein are those remaining after subtraction of GFP counterpart interactors. According to their cellular function, most of the proteins shown in this network were classified into two general groups: spliceosome (top right image) and ribosome (lower left image).

Total extract from GFP-DNAC17 cells were used to identify DNAJC17 interacting proteins by Co-immunoprecipitation (Co-IP) experiments followed by mass spectrometry analysis. GFP cells were used as negative control in parallel experiments. Total protein extracts from GFP-DNAJC17 and GFP cells were immunoprecipitated by using GFP trap beads, digested with trypsin and analyzed by nanoLC-ESI-LIT-MS/MS. Thus, mass spectrometry analysis identified 70 proteins uniquely observed in the GFP-DNAJC17 sample, when compared with the GFP counterpart (Table S2). These results allowed describing the Interactome Network of DNAJC17, which is shown in Fig. 3. Classification of the interacting proteins by the Kyoto Encyclopedia of Genes and Genomes (KEGG) database (http://www.genome.jp/kegg/) identified two general groups of cellular functions related to RNA metabolism: the one involving components related to the spliceosome machinery and the one including species involved in ribosome structure/function.

### Validation and *in vivo* interaction of DNAJC17 with spliceosomal proteins

**Figure 4:**
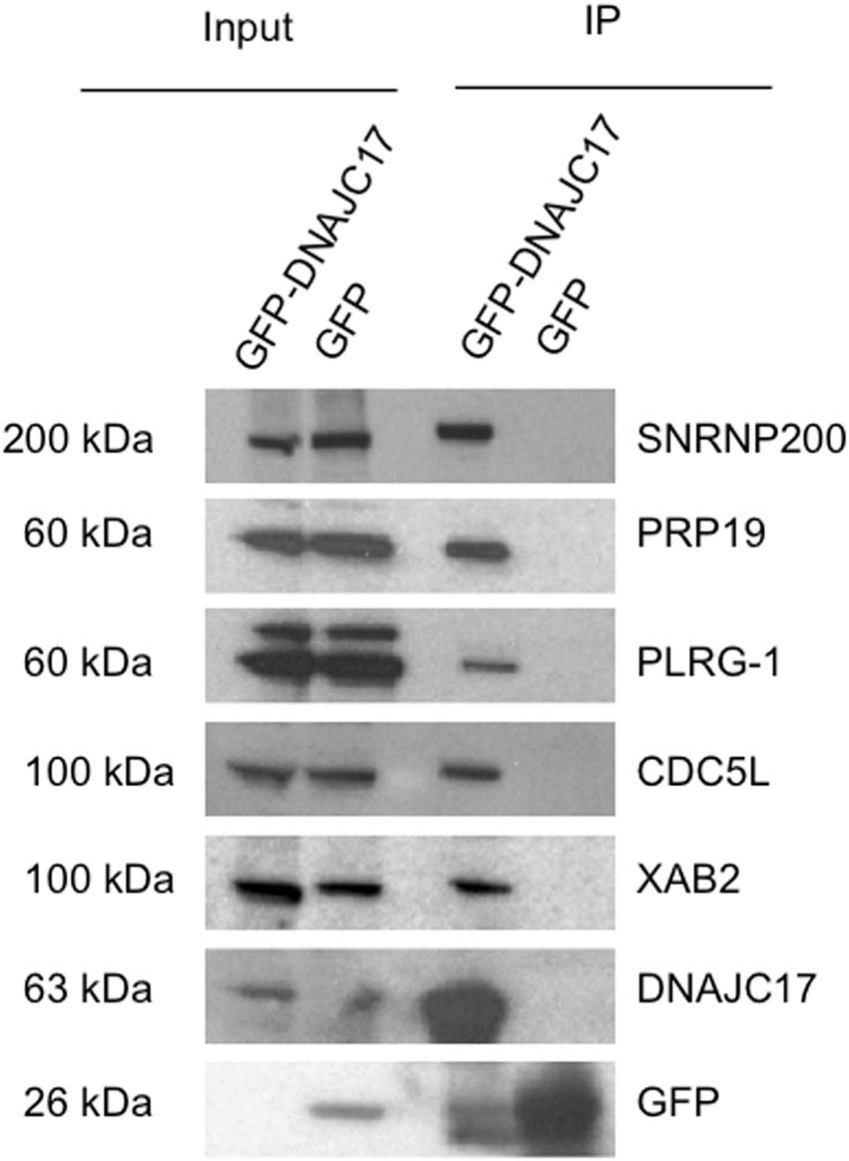
Validation of DNAJC17-interacting partners. Lysates from GFP-DNAJC17 and GFP cells were subjected to co-immunoprecipitation analysis with GFP-Trap®_A affinity beads (Chromotek). Immunoprecipitates were analyzed by Western blotting with the antibodies indicated on the right of each image. The image shows that DNAJC17 co-immunoprecipitates with SNRNP200, PRP19, PLRG-1, CDC5L and XAB2. Molecular mass of each detected protein is indicated on the left.

To validate the reliability of the ascertained DNAJC17 interactomic data, we selected PRP19, PLRG1, SNRNP200 and XAB2 because their knockout in mouse causes early embryo lethality^15–17^ (International Mouse Phenotyping Consortium IMPC MP: 0013292 www.mousephenotype.org) like that of DNAJC17^3^. Interestingly, all four selected proteins play a role in pre-mRNA splicing (Table 1). We also selected CDC5L protein, whose knockout is not reported in literature, but it is known to interact with PRP19 e PLRG-1^18^. Relevant informations about the selected interactors are reported in Table 1. Total protein extracts from GFP-DNAC17 cells were immunoprecipitated by using GFP trap beads to capture GFP-DNAJC17-associated protein complexes (for details see Materials and Methods). Immunoprecipitates were then resolved by western blot confirming that DNAJC17 interacts with PRP19, PLRG1, CDC5L, SNRNP200 and XAB2 proteins (Fig. 4**)**.

**Table 1:** Proteins selected for validation. Four proteins from proteomic analysis were identified as splicing factors essential for embryogenesis, namely SNRNP200, PRP19, PLRG-1 and XAB2. CDC5L belongs to the Prp19/CDC5L complex.

UniProt #	Gene name	Function	Localization	Knockout mice	References	Human diseases
O75643	SNRNP 200	Prp19/CDC5L complex	Nucleus	Embryonic lethality before implantation	J:204739 International Knockout Mouse Consortium	Retinitis pigmentosa 33 (Zhao *et al*., 2009)
Q9UMS4	PRP19	Prp19/CDC5L complex	Nucleus	Embryonic lethality before implantation	Fortschegger *et al*., 2007	
A8MW61	PLRG-1	Prp19/CDC5L complex	Nucleus	Embryonic lethality before implantation	Kleinridders *et al*., 2009	
B4DSH1	CDC5L	Prp19/CDC5L complex	Nucleus	—		Bilateral multicystic renal dysplasia (Groenen *et al*., 1998)
Q68CN2	XAB2	Splicing factor	Nucleus	Embryonic lethality before implantation	Yonemasu R *et al*. 2005

**Figure 5:**
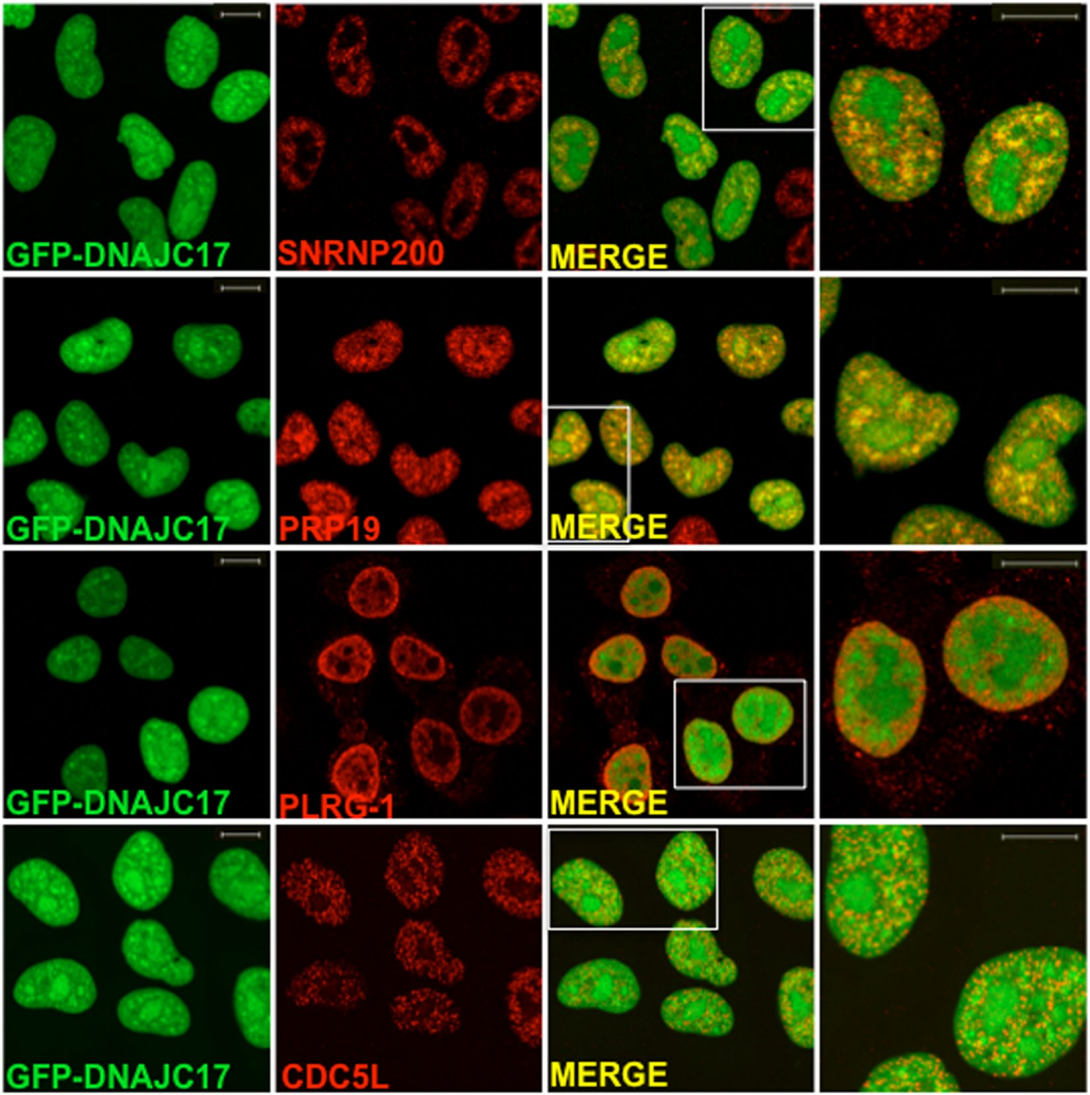
Confocal analysis of GFP-DNAJC17 cells stained with PRP19, CDC5L and SNRNP200 antibodies. GFP-DNAJC17 cells were induced with tetracycline for 16 h. Endogenous GFP-DNAJC17 fluorescence is shown in green. Staining with anti-PRP19, anti-CDC5L, anti-SNRNP200 or anti-PLRG1 is shown in red. The last column shows a detail of the merged image (right panel). Co-localization is represented with increasing intensity of yellow in the merged images and their details (scale bar 10 µm).

To test if such interactions take place also *in vivo*, subcellular co-localization of DNAJC17 with its candidate interactors was then analyzed by immunofluorescence in fixed cells. As it is shown in Fig. 5, co-localization of DNAJC17 with SNRNP200 and PRP19 was more evident respect to that with PRLG-1 and CDC5L. To measure subcellular co-localization, confocal images were analyzed with the Image J software using the JACoP plugin calculating the Pearson’s coefficient and following the Coste’s approach^19^. Immunofluorescence assay indicated that DNAJC17 co-localizes with PRP19 (mean Pearson’s coefficient: 0.805 ± 0.057; Coste’s randomization P value: 100%), PLRG1 (mean Pearson’s coefficient: 0.739 ± 0.050; Coste’s randomization P value: 100%), CDC5L (mean Pearson’s coefficient: 0.571 ± 0.037; Coste’s randomization P value: 100%), SNRNP200 (mean Pearson’s coefficient: 0.651 ± 0.057; Coste’s randomization P value: 100%). Due to technical problems, we were unable to confirm the XAB2 co-localization. To further confirm the co-localization of DNAJC17 with two candidate interactors (PLRG-1 and SNRNP200), we performed double immunofluorescence also in parental HeLa cells by detecting the endogenous protein with a specific antibody^3^ (Figure S1). These data confirmed that DNAJC17 shares subcellular compartment with spliceosomal proteins, thus suggesting a possible role of DNAJC17 in modulating spliceosomal activity.

### DNAJC17 localizes at nuclear speckles

**Figure 6:**
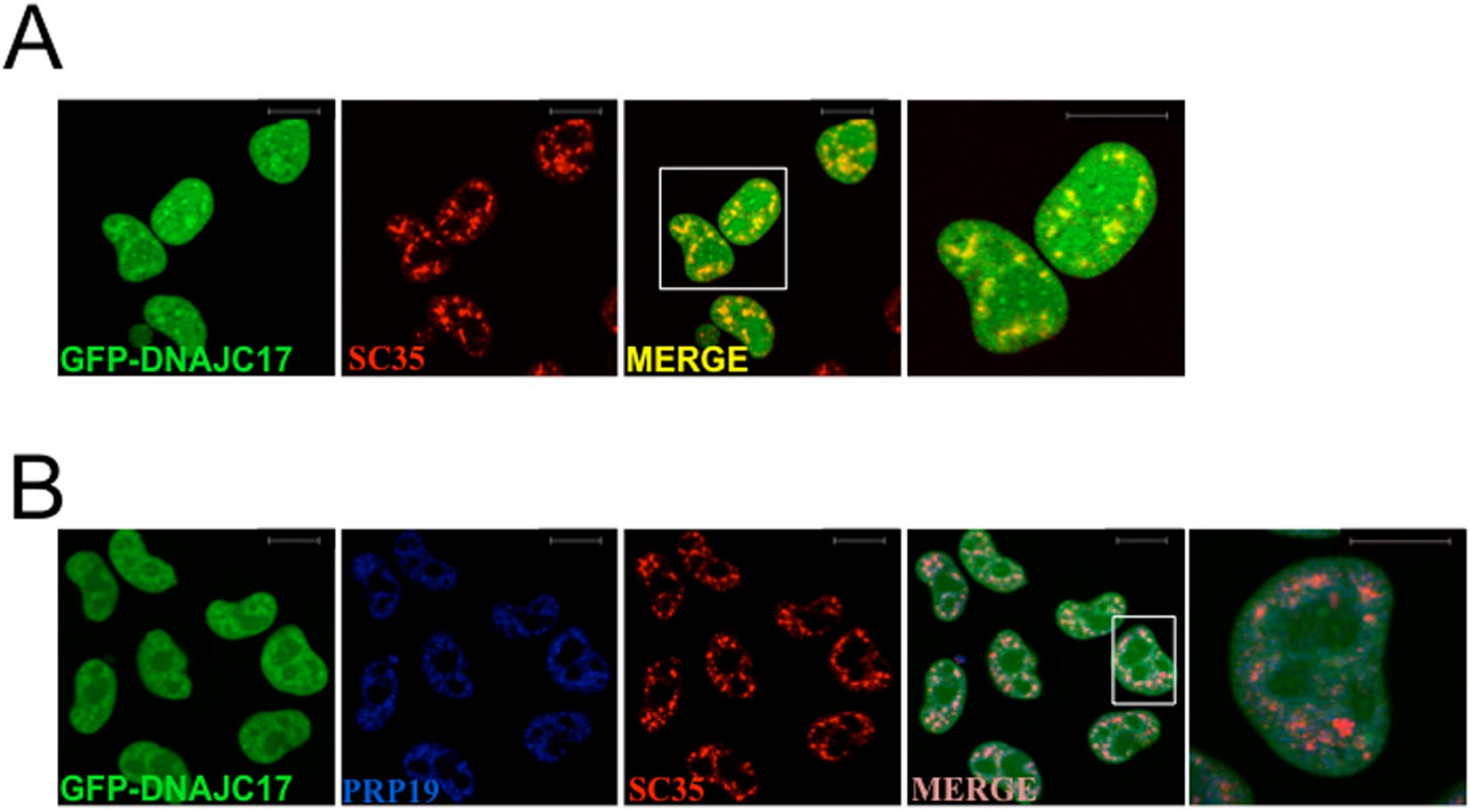
Confocal analysis of GFP-DNAJC17 cells stained with PRP19 and SC35 antibodies. GFP-DNAJC17 cells were induced with tetracycline for 16 h. Endogenous GFP-DNAJC17 fluorescence is shown in green. (**A**) Staining with anti-SC35 is shown in red. The last panel shows a detail of the merged image (right image). Co-localization is represented with increasing intensity of yellow in the third and last images (**B**). Anti-SC35 staining is shown in red, while the anti-PRP19 is shown in blue. Co-localization is represented with increasing intensity of pink in the fourth and last images (scale bar 10 µm).

Based on its interaction with PRP19, PLRG1, CDC5L and SNRNP200 and the speckled pattern of nuclear localization (Fig. 5), we tested if DNAJC17 interaction with its partners occurs at nuclear speckles, where all these proteins reside. To this purpose, we checked DNAJC17 co-localization with a specific marker of such compartment, namely SC35, a splicing factor of the serine-rich (SR) family of proteins generally used to label splicing speckles^20–22^. Immunofluorescence analysis showed that the nuclear dots of DNAJC17 clearly overlapped with SC35 staining (Fig. 6A), thus demonstrating that DNAJC17 localizes at splicing speckles (mean Pearson’s coefficient: 0.648 ± 0.035; Coste’s randomization P value: 100%). As PRP19 and CDC5L interact within speckles, we also verified whether DNAJC17 co-localizes with such complex in the same compartment. Double immunofluorescence analysis of GFP-DNAJC17 cells with PRP19 and SC35 antibodies indicated a high co-localization index for the three proteins (mean Pearson’s coefficient: 0.722 ± 0.052; Coste’s randomization P value: 100%) (Fig. 6B).

### DNAJC17 modulates splicing efficiency

**Figure 7:**
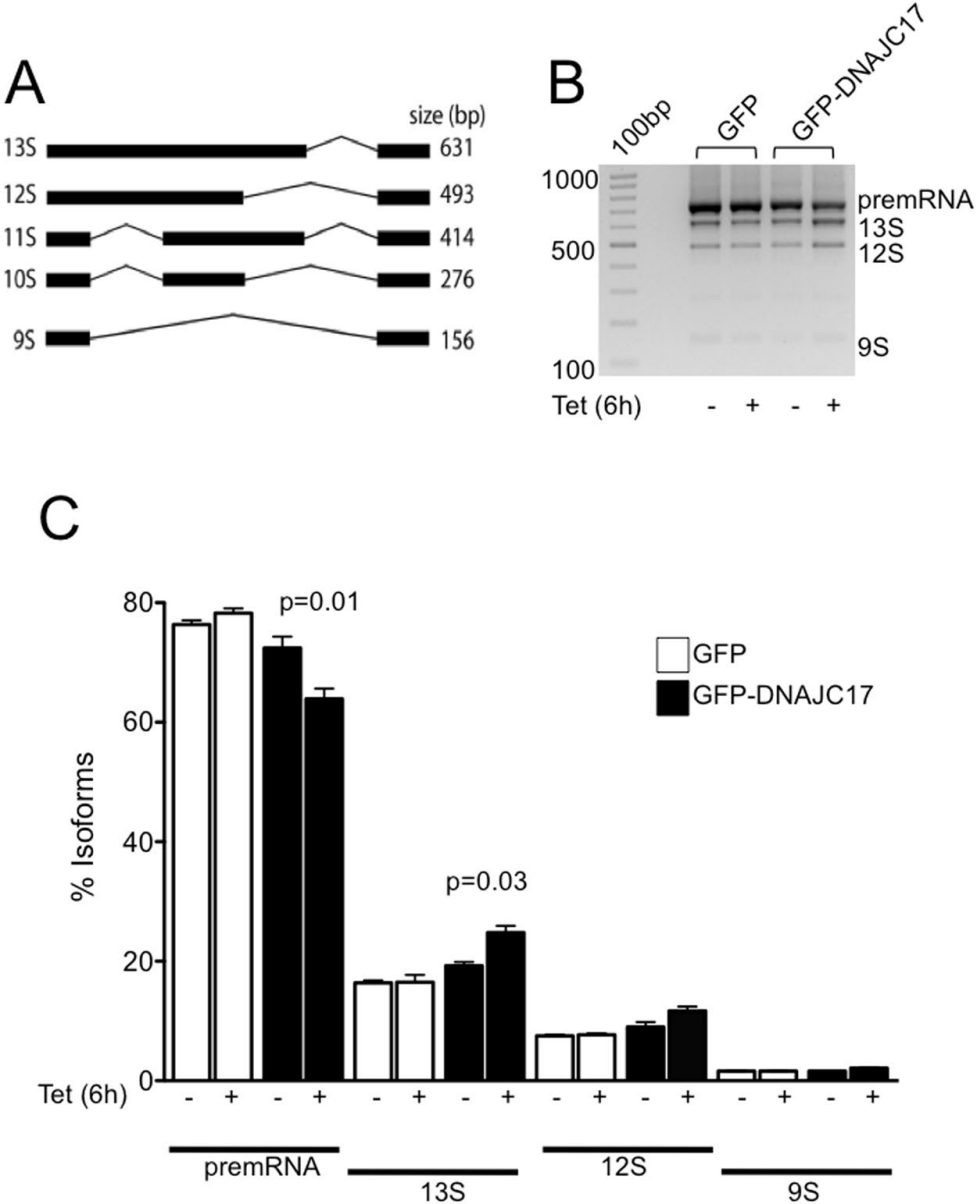
DNAJC17 regulates RNA splicing. (**A**) Schematic representation of E1A-derived splicing variants. (**B)** Splicing of E1A was analyzed by RT–PCR in E1A minigene-transfected cells. GFP cells (lanes 1 and 2) and GFP- DNAJC17 cells (lanes 3 and 4) were untreated or treated with Tet for 6 h. Splicing products are indicated on the right of the panel. (**C**) Quantification of the major E1A mRNA variants: white bars and black bars represent the percentage of each isoform in GFP and GFP-DNAJC17 cells, respectively, either untreated or treated with Tet for 6 h. Data represent the mean ± SD of three independent experiments.

The presence of a RRM motif in its structure, as well as its interaction/localization with splicing factors in nuclear speckles, suggested a possible role of DNAJC17 in the modulation of the splicing activity. To test whether DNAJC17 modulates alternative splicing, GFP-DNAJC17, GFP and parental HeLa cells were used to perform *in vivo* splicing assays using the E1A minigene, a commonly used splicing target containing several 5′ and 3′ alternative splice sites (Fig. 7A)^23^. In GFP-DNAJC17 cells transfected with E1A for 42 h and treated with Tet for 6 h, we observed a perturbation of E1A minigene splicing (Fig. 7B,C). In particular, we observed a reduction of the unspliced pre-mRNA and a concomitant increase in splicing efficiency of the 13 S variant (Fig. 7B,C), which utilizes the proximal 5′ splice site. In contrast, no significant changes of the 12 S and 9 S alternative splice variants were observed (Fig. 7C). These results indicated that DNAJC17 is able to modulate E1A minigene splicing.

**Figure 8:**
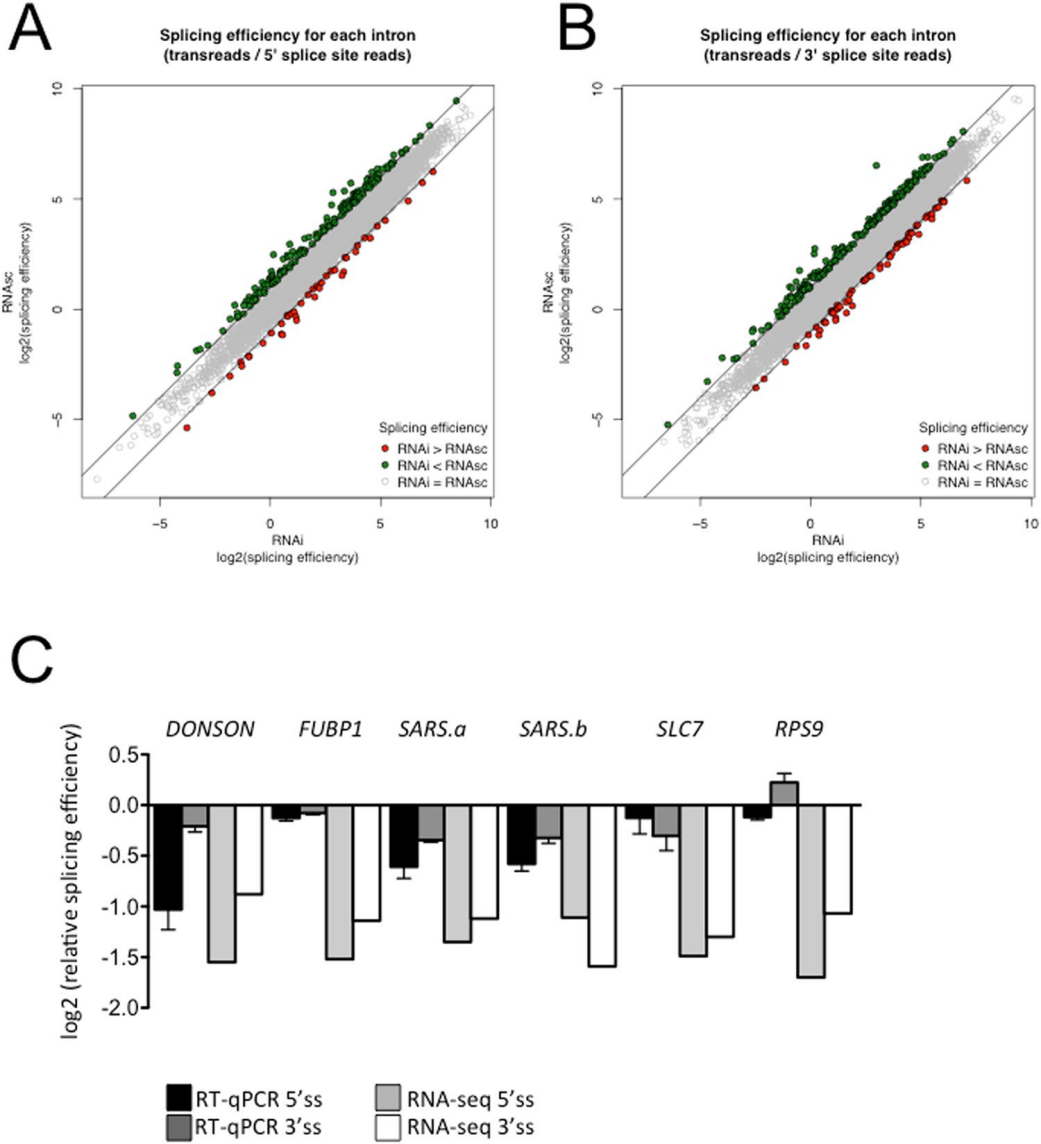
Splicing efficiency in DNAJC17_kd_. The scatter plots represents for each intron 5′ (**A**) and 3′ (**B**) splicing efficiency in DNAJC17_kd_ (x axis) and in the control DNAJC17_wt_ (y axis), as the mean across the three replicates. Considering a cutoff of absolute values ≥1, introns whose splicing efficiency is reduced following DNAJC17 silencing are labeled in green; instead red indicates increased efficiency compared with control. (**C**) Comparison of relative splicing efficiencies at the 5′ and 3′ ends of selected introns calculated from the RNA-seq data with corresponding splicing efficiencies as determined by RT-qPCR (means of 3 independent RT-qPCR experiments ± SD in triplicates). For each intron, ratio between exon-exon junction transreads and intron 5′ or 3′ ends in DNAJC17_kd_ cells are reported relative to DNAJC17_wt_ control.

To further corroborate the hypothesis of a role of DNAJC17 in splicing, we re-analyzed RNAseq data obtained in DNAJC17_kd_ cells using a recently published pipeline designed for determination of pre-mRNA splicing efficiency in yeast^24^. This workflow calculates splicing efficiency values separately for the 5′ and 3′ splice junctions of each intron, as the ratio between the number of exon-exon junction-spanning reads (transreads) and the number of reads covering the first base of 5′ intron end (5′ efficiency) or the last base of 3′ intron end (5′ efficiency) (Table S3). We considered only splicing efficiency changes whose absolute values of ratios were ≥1 (Fig. 8A,B**)**. While the majority of introns show unaltered splicing in DNAJC17_kd_ cells (Fig. 8A,B grey dots), there are specific genes displaying increased or decreased intron retention (Fig. 8A,B green or red dots, respectively). It is noteworthy that the number of transcripts with increased intron retention (in green) is higher respect to that with decreased retention (in red), and also their extent of variation showed higher values (Table S3). To validate the analysis, we measured by RT-qPCR intron retention ratio of five genes (*DONSON*, *FUBP1*, two different introns of *SARS*, *SARS*.*a* and *SARS*.*b*, *SLC7* and *RPS9*) showing various degrees of splicing impairment in DNAJC17_kd_ cells (Table S4). Splicing efficiencies measured by RT-qPCR show similar trends respect to those calculated using RNAseq datasets (Fig. 8C).

## Discussion

Notwithstanding previous studies that identified *Dnajc17* as a susceptibility gene for congenital hypothyroidism^2^ and demonstrated its wide expression in mouse since early embryogenesis, in which it plays an essential role in pre-implantation stages^3^, the molecular function of DNAJC17 protein is still elusive. In this study, a multi-omic approach was undertaken to address this issue, which was complemented with additional localization and functional experiments. In particular, transcriptomic analysis of DNAJC17-depleted cells showed that hampering of *Dnajc17* expression affected the level of structural genes involved in gene expression processes, mainly related to tRNA and amino acid modifications. These results suggest that DNAJC17 may be involved in one (or more) gene expression-related cellular function(s).

On the other hand, proteomic experiments aimed at defining the DNAJC17 protein interactome identified 70 proteins as selectively interacting with DNAJC17. Most of these interactors fall into two protein functional groups: i) spliceosome components/interactors; ii) ribosome structural proteins. Some of these candidates were validated by independent co-IP/western blotting experiments. Both identified protein networks are involved in RNA metabolism, suggesting once again that DNAJC17 may be implicated in gene expression. The presence in the DNAJC17 protein interactome of a huge number of factors involved in splicing-related processes suggested that DNAJC17, similarly to its ortholog Cwc23 in yeast^9^, may play a role in pre-mRNA splicing. Interestingly, several candidate interactors reported in this study have already been demonstrated to play an essential role in mouse development, similarly to DNAJC17^3^. This is the case of 4 DNAJC17-binding partners, namely PRLG1, PRP19, SNRNP200 and XAB2. Among that, PRLG1 and PRP19, together with another protein identified as a DNAJC17 interactor, namely CDC5L, have been demonstrated forming a complex that plays a key role in catalytic activation of the spliceosome^18,25^. Furthermore, knock-out mice for *Prlg1* and *Prp19* showed embryo lethality at the first cell division and blastocyst stage, respectively^15,16^. The interaction and co-localization of DNAJC17 with CDC5L, PRLG1 and PRP19 was confirmed by both co-IP and immunofluorescence assays. Such interaction with the core of the PRP19/CDC5L complex suggested a possible involvement of DNAJC17 in the correct formation and/or function of the latter molecular machinery. A similar function was also hypotesized for SNRNP200, a U5 small nuclear ribonucleoprotein (snRNP) helicase that is required for spliceosome assembly, and whose depletion blocks the second step of splicing^26^. On the other hand, it was observed that the homozygous knockout of SNRNP200 causes embryonic lethality before implantation (International Mouse Phenotyping Consortium IMPC MP: 0013292 www.mousephenotype.org).

Based on the ascertained interaction of DNAJC17 with several proteins involved in splicing, we further hypotesized that such interaction could take place within nuclear speckles. These are indeed discrete nuclear domains where pre-messenger RNA, splicing factors, snRNP particles, spliceosome subunits and non-snRNP protein splicing factors accumulate. In particular, it is well accepted that nuclear speckles act as storage compartments able supplying splicing factors to active transcription sites^20–22^. In this study, we were able to demonstrate that DNAJC17 co-localizes with SC35, a well-established marker of localization in nuclear speckles, thus demonstrating that this chaperone is present in these subnuclear structures.

Localization in nuclear speckles, together with the association with many splicing proteins, was strongly suggestive for a role of DNAJC17 in splicing, likely to its yeast ortholog Cwc23^9^, whose function was hypothesized based on experiments with gene deletion mutants. In agreement with this hypothesis, E1A minigene assays demonstrated that DNAJC17 overexpression in HeLa cells was able to modulate splicing efficiency. Moreover, by re-analyzing DNAJC17_kd_ RNAseq data trough a workflow specifically designed for splicing efficiency analysis, we highlighted few hundreds of introns differentially retained in DNAJC17_kd_ respect to DNAJC17_wt_ cells. Hence, our data strongly suggest that DNAJC17 plays a modulatory role in splicing mechanisms, which hence appears to be evolutionary conserved moving from yeast to human. Molecular chaperones have already been shown to control splicing processes, both in yeast^9^ and in human cells, although in these latters only chaperones controlling cytoplasmic steps of spliceosome assembly have been identified^27^. In this paper we report the first evidence of interaction of a HSP40 protein with spliceosomal core components in the nucleus.

It is worth noting that homozygous truncating mutations of DNAJC17 in humans segregate with a novel syndromic form of retinitis pigmentosa (RP), which is associated with hypergammaglobulinemia^4^. RP is a genetically heterogeneous disorder, however recent evidences have pointed out that splicing defects due to mutations of several different genes are at the basis of the pathogenesis of such disease^28^.

Taken together, our data represent the first functional characterization of human DNAJC17; they strongly suggest that this protein is involved in splicing events, although its exact role in this process remains to be defined. The identification of numerous splicing-related interactors will allow to test if DNAJC17 is able to modulate their activity by acting as a chaperone. Thus, further studies are required to establish whether DNAJC17 plays a role in general or specific alternative splicing events. Nevertheless, its involvement in such a crucial phenomenon should hypothetically explain the embryo lethality in DNAJC17 knockout mouse as well as the association of different mutations in Dnajc17 to different complex disorders in humans^4,5,7^.

## Methods

### Generation of Stable HeLa Cell Lines

DNAJC17 cDNA from Sv129 mouse strain previously cloned^3^ was amplified by PCR with primers containing BamHI and XhoI restriction sites (DnajC17 Bamh1Fw: 5′gagctcggatccatggcggtgaccaaagagctctt3′; DnajC17Xho1Rev: 5′ctctagactcgagctacgtgggccgcccctc3′). The resulting PCR product was digested and cloned into a modified pCDNA5/FRT/TO (Invitrogen, BioSource International, USA) in which the GFP coding sequence was cloned upstream the multiple cloning sites^29^. The construct was sequenced to verify the absence of mutations. pCDNA5/FRT/TO containing GFP or chimeric GFP- DNAJC17 was transfected in Flp-In T-REx HeLa cells (Invitrogen, BioSource International, USA) by using the Effectene Transfection Reagent (Qiagen, Milan, Italy) and the positive cells were selected according to the manufacturer’s instructions (Invitrogen K6500-01). After the selection, GFP or GFP-DNAJC17 expression was induced with tetracycline, and fluorescent cells were sorted using a MoFlo sorter (Beckman Coulter, Milano, Italy). GFP or GFP- DNAJC17 fusion protein expression was tested by western blot before and after tetracycline induction.

### RNA sequencing

Differential gene expression was analysed in DNAJC17-interfered HeLa cells by a RNA sequencing (RNA-Seq) approach. RNA was extracted from 3 independent samples for both knockdown and wild type conditions; it was sequenced on HiSeq. 1500 (Illumina, San Diego, CA, USA) according to manufacturer’s protocols. About 7 × 10^7^ high quality sequence reads were produced for sample with a mean quality score ≥30 (99.9% base quality accuracy). The human Ensembl GRCh37 genome was used as reference for read mapping, which was performed by TopHat aligner^11^. Properly mapped reads (about 5 × 10^7^) were further processed using Cufflinks package^12^. Selected reads were assembled into transcripts, which were compared among the six samples and with known annotations to generate a unique final transcriptome. Resulting 20825 genes were then tested for differential expression between DNAJC17_kd_ vs DNAJC17_wt_ and filtered for corrected p-value (FDR) ≤0.05 and absolute fold change ≥1.5 (884 significantly deregulated genes; 360 up-regulated and 524 down-regulated genes).

RNAseq aligned reads were further analyzed to calculate the splicing efficiency in the two conditions, using the method described by Prevorovsky *et al*.^24^. Briefly, the splice sites were predicted through the analysis of the reads spanning the exons junctions (setting the minimum and maximum intron length to 20 and 11000 bases, respectively). Then, we calculated the splicing efficiency for each single intron and separately for the 5′ and 3′ splice site, as the ratio between the number of spanning reads and the number of reads covering the first base of 5′ intron end (5′ efficiency) or the last base of 3′ intron end (5′ efficiency).

### Immunoblotting

Whole-cell lysates of HeLa, GFP-DNAJC17 and GFP cells were prepared in sample buffer (1% w/v Triton X-100, 0.1% w/v SDS, 0.50% w/v sodium deoxycholate, 50 mM TrisHCl pH 8.5X mM MgCl_2_, 150 mM NaCl, 1 mM DTT, 0.5 PMSF, X w/v Protease inhibitor cocktail, 50 mM sodium fluoride, 0.5 mM sodium pyrophosphate and 0.5 mM sodium orthovanadate) and normalized for protein concentration by Pierce BCA Protein assay kit (Thermo Scientific, Milano, Italy). A 20 μg amount of protein samples was resolved on a precast NuPAGE 4–12% Bis-Tris Gel (Life Technologies, Monza, Italy) and transferred on a polyvinylidene difluoride (PVDF) membrane (Millipore, Milano, Italy). Nonspecific binding sites were blocked by incubation with 5% w/v non-fat dry milk in TBS (20 mM TrisHCl pH 7.6, 140 mM NaCl) containing 0.1% w/v Tween 20. Immunodetection was performed by using one of the following primary antibodies: DNAJC17 home-made rabbit polyclonal antibodies^3^; GFP monoclonal antibody (Chromotek, Planegg-Martinsried, Germany); PRP19 polyclonal antibody (LifeSpan BioSciences, Seattle, WA, USA); CDC5L monoclonal antibody (Santa Cruz Biotechnology, Dallas, Usa); PLRG1 polyclonal antibody (Novus Biologicals, Littleton, CO, USA); HELIC2 (SNRNP200) polyclonal antibody (Santa Cruz Biotechnology, Dallas, Usa); SC35 monoclonal antibody and α1 tubulin monoclonal antibody (Sigma-Aldrich, St. Louis, MO, USA). Immune complexes were detected by enhanced chemiluminescence as instructed by the manufacturer (Pierce, Thermo-Fisher, San Jose, CA, USA) and captured by SuperRX FUJI medical X-ray film (FUJIFILM Corp. Tokio, Japan). Digital images of films were obtained by ChemiDoc XRS with ImageLab software (Bio-Rad, Hercules, CA, USA).

### Co-immunoprecipitation

GFP-DNAJC17 fusion protein or GFP protein expressions were induced with tetracycline (2 µg/ml) overnight, according to manufacturer’s instructions (Invitrogen, BioSource International, USA, K1020). Four confluent 15-cm plates of DNAJC17-GFP or GFP were harvested in PBS, spin down at 1000 g for 5 minutes and suspended in 1 mL of lysis buffer (50 mM Tris pH 7.4, 150 mM NaCl, 10 mM EDTA, 2 mM MgCl_2_, 0.1% w/v Triton X100). After incubation on ice for 30 min, extracts were passed several times through a 20 g 1 and ½ needle. Protein extracts were clarified by centrifugation at 13000 g for 10 min, and then transferred in clean tubes. Ten µL of GFP trap (Chromotek, Planegg-Martinsried, Germany gta-20) were added to the protein extract and the tubes were incubated under rotation for 2 h, at 4 °C. GFP trap beads were pulled-down at 1000 g for 5 min, and washed 5 times with washing buffer (50 mM Tris pH 7.4, 200 mM NaCl, 10 mM EDTA, 2 mM MgCl_2_, 0.1% w/v Triton X100). Then, the washes beads were boiled in 50 µL of 1X LDS (Invitrogen, BioSource International, USA NP0008), containing 1X sample reducing agent (Invitrogen, BioSource International, USA NP0004). Five µL of each sample eluate were separated on 4–12% bis-Tris acrylamide gel electrophoresis (Invitrogen, BioSource International, USA NP0321BOX) and then silver stained; the remaining 45 µL were used for mass spectrometry-based protein identification.

### In gel digestion, mass spectrometry analysis and protein identification

Co-immunoprecipitation eluates from GFP-DNAJC17-GFP and GFP samples were resolved by 12% SDS-PAGE and then stained with colloidal Coomassie G-250^30^; gel image was acquired by using an Image Scanner III densitometer (GE Healthcare Life Sciences, Milano, Italy). Each sample was manually cut into 23 slices, which were excised from gel, triturated and washed with water. Proteins were *in-gel* reduced, S-alkylated and digested with trypsin as previously reported^31^. Digest aliquots were removed and subjected to a desalting/concentration step on ZipTip μC18 disk (Millipore, Milano, Italy) using 5% formic acid/50% acetonitrile as eluent before nanoLC-ESI-LIT-MS/MS analysis. Digests were analyzed by nanoLC-ESI-LIT-MS/MS using a LTQ XL mass spectrometer (Thermo Finnigan, San Jose, CA, USA) equipped with Proxeon nanospray source connected to an Easy-nanoLC (Proxeon, Odense, Denmark). Peptide mixtures were separated on an Easy C18 column (100 × 0.075 mm, 3 μm) (Proxeon, Odense, Denmark) by using a linear gradient of acetonitrile containing 0.1% formic acid (solvent B) in aqueous 0.1% formic acid (solvent A); solvent B ramped from 5% to 40% over 60 min, and from 40% to 80% in 10 min, at a flow rate of 300 nL/min. Spectra were acquired within the range *m/z* 400–2000. Acquisition was controlled by a data-dependent product ion scanning procedure over the three most abundant ions, enabling dynamic exclusion (repeat count 1 and exclusion duration 1 min). The mass isolation window and collision energy were set to *m/z* 3 and 35%, respectively. Duplicate analysis of each sample was performed.

Proteome Discoverer 1.3 platform (Thermo-Fisher, San Jose, CA, USA) was used to search raw mass data against an updated human UniprotKB non-redundant protein sequence database (76058 protein sequences, 2016) with MASCOT (Matrix Science, UK) algorithms. Database searching was performed by using a mass tolerance value of 2.0 Da for precursor ion and 0.8 Da for MS/MS fragments, trypsin as proteolytic enzyme, a missed cleavages maximum value of 2 and Cys carbamidomethylation as fixed modification, and Met oxidation and pyro-Glu (N-terminal Gln) as variable modifications, respectively. Candidates with more than 2 assigned unique peptides with an individual MASCOT score > 25 were further considered for protein identification.

### Immunofluorescence assays

Cells were fixed with 3.7% paraformaldehyde and permeabilized with PBS containing 0.1% w/v Triton X-100. For protein detection, the primary antibodies used have been already decribed in the Immunoblotting section. Secondary antibodies were mouse, human, rabbit or goat IgG conjugated to a fluorochrome (Alexa Fluor 448, 555 and 647, ThermoFisher Scientific, Milano, Italy). Antibody incubations were performed in PBS containing 2% w/v BSA and 0.1% w/v Triton X-100, for 1 h, at room temperature. Coverslips were mounted with mounting medium for fluorescence with DAPI (Vectashield, Vector Laboratories, Burlingame, CA, USA). Confocal microscopy and overlay analysis were performed on a Zeiss LSM510 Confocal Laser Scanning Microscope with an x63 objective.

### Confocal microscopy image analysis

Co-localization analysis was performed using JACoP plug-in embedded in the visualization and analysis software ImageJ^19^. Analysis was performed on a similar-sized symmetrical region of interest (ROI) selected for each dye. Each coloured image was split into respective red, green, and blue channels. The comparative degree of co-localization was calculated as mean Pearson’s R coefficients and Coste’s randomization P value on the red and green channels, using the embedded co-localization analysis plug-in at default settings.

### RNA interference

The HeLa cell line was grown in Dulbecco’s modified Eagle medium (Euroclone, Milano, Italy) supplemented with 10% v/v fetal calf serum (Hyclone, GE Healthcare Life Sciences, Milano, Italy). Cells were plated (18*10^4^) in a 6 well plate in antibiotic-free complete medium and transfected the following day with 50 nM DNAJC17 siRNA (ON-TARGETplus Human DNAJC17 siRNA, cat n° L-021141, Dharmacon, GE Healthcare Life Sciences, Milano, Italy) and 50 nM siRNA negative control (ON-TARGETplus Non-targeting Pool, cat n° # D-001810 Dharmacon, GE Healthcare Life Sciences, Milano, Italy) in INTERFERin transfection reagent (cat n° 409-10 Polyplus-transfection, Illkirch FRANCE) following the manufacturer’s protocol; three technical replicates were prepared for each condition. Cells were harvested 96 h after transfection and total RNA was extracted with TRIzol reagent (Thermo-Fisher, San Jose, CA, USA**)** for RNA-seq transcriptome analysis.

### RT-qPCR Analysis

cDNA was generated with the SuperScript® III First-Strand Synthesis System for RT-PCR (Invitrogen 18080051), according to the manufacturer’s specifications. Six differentially retained introns belonging to five genes were selected for validation. For each intron, three primer pairs were designed to specifically amplify either the spliced exon-exon (transread), the unspliced 5′ exon-intron (5′ ss) and 3′ intron-exon (3′ ss) junctions. Primers sequence are reported in Table S4. Real-time PCR on total cDNA was performed with iTaq™ Universal SyBR® Green Supermix (Bio Rad 172-5124) using gene-specific oligos. Relative amplicon amounts were calculated by the ΔΔCt method^32,33^. Comparison of data sets was performed by Student’s t-test and a value of p < 0.05 was considered signicant.

### E1A splicing assay

GFP-DNAJC17, GFP and HeLa cells were transfected with E1A minigene^23^ using Lipofectamine 2000 (Invitrogen, BioSource International, USA), according to manufacturer’s instruction. After 42 h of transfection, tetracycline (2 mg/ml) was added to the medium, and cells were collected 6 h later for RNA and protein extraction. One mg of total DNA-free RNA was used for cDNA generation by using M–MLV reverse transcriptase (Promega, Milano, Italy). RT-PCR analysis was performed as previously described^23^. Equal amount of RT-PCR reactions were run on agarose gel and digital images were captured and analyzed by ChemiDoc XRS with ImageLab software (Bio-Rad, Hercules, CA, USA).

### Data availability statement

The datasets and reagents generated and/or analysed during the current study are available from the corresponding authors on reasonable request.

## Electronic supplementary material

Figure S1

Table S1

Table S2

Table S3

Table S4

## References

[1] Kampinga HH, Craig EA (2010). The HSP70 chaperone machinery: J proteins as drivers of functional specificity. Nat Rev Mol Cell Biol.

[2] Amendola E (2005). A mouse model demonstrates a multigenic origin of congenital hypothyroidism. Endocrinology.

[3] Amendola E (2010). A locus on mouse chromosome 2 is involved in susceptibility to congenital hypothyroidism and contains an essential gene expressed in thyroid. Endocrinology.

[4] Patel N (2016). Expanding the clinical, allelic, and locus heterogeneity of retinal dystrophies. Genet Med.

[5] Al Assaf C (2014). Screening of JAK2 V617F and MPL W515 K/L negative essential thrombocythaemia patients for mutations in SESN2, DNAJC17, ST13, TOP1MT, and NTRK1. Br J Haematol.

[6] Hou Y (2012). Single-cell exome sequencing and monoclonal evolution of a JAK2-negative myeloproliferative neoplasm. Cell.

[7] Stamova BS (2013). Evidence for differential alternative splicing in blood of young boys with autism spectrum disorders. Mol Autism.

[8] Pandit S (2009). Spp382p interacts with multiple yeast splicing factors, including possible regulators of Prp43 DExD/H-Box protein function. Genetics.

[9] Sahi C, Lee T, Inada M, Pleiss JA, Craig EA (2010). Cwc23, an essential J protein critical for pre-mRNA splicing with a dispensable J domain. Mol Cell Biol.

[10] Long JC, Caceres JF (2009). The SR protein family of splicing factors: master regulators of gene expression. Biochem J.

[11] Trapnell C, Pachter L, Salzberg SL (2009). TopHat: discovering splice junctions with RNA-Seq. Bioinformatics.

[12] Trapnell C (2010). Transcript assembly and quantification by RNA-Seq reveals unannotated transcripts and isoform switching during cell differentiation. Nat Biotechnol.

[13] Yu G, Wang LG, Han Y, He Q (2012). Y. clusterProfiler: an R package for comparing biological themes among gene clusters. OMICS.

[14] Craig EA, Marszalek J (2017). How Do J-Proteins Get Hsp70 to Do So Many Different Things?. Trends Biochem Sci.

[15] Fortschegger K (2007). Early embryonic lethality of mice lacking the essential protein SNEV. Mol Cell Biol.

[16] Kleinridders A (2009). PLRG1 is an essential regulator of cell proliferation and apoptosis during vertebrate development and tissue homeostasis. Mol Cell Biol.

[17] Yonemasu R (2005). Disruption of mouse XAB2 gene involved in pre-mRNA splicing, transcription and transcription-coupled DNA repair results in preimplantation lethality. DNA Repair (Amst).

[18] Grote M (2010). Molecular architecture of the human Prp19/CDC5L complex. Mol Cell Biol.

[19] Bolte S, Cordelieres FP (2006). A guided tour into subcellular colocalization analysis in light microscopy. J Microsc.

[20] Hall LL, Smith KP, Byron M, Lawrence JB (2006). Molecular anatomy of a speckle. Anat Rec A Discov Mol Cell Evol Biol.

[21] Handwerger KE, Gall JG (2006). Subnuclear organelles: new insights into form and function. Trends Cell Biol.

[22] Lamond AI, Earnshaw WC (1998). Structure and function in the nucleus. Science.

[23] Yang X (1994). The A1 and A1B proteins of heterogeneous nuclear ribonucleoparticles modulate 5′ splice site selection *in vivo*. Proc Natl Acad Sci USA.

[24] Prevorovsky M, Halova M, Abrhamova K, Libus J, Folk P (2016). Workflow for Genome-Wide Determination of Pre-mRNA Splicing Efficiency from Yeast RNA-seq Data. Biomed Res Int.

[25] Makarov EM (2002). Small nuclear ribonucleoprotein remodeling during catalytic activation of the spliceosome. Science.

[26] Umen JG, Guthrie C (1995). The second catalytic step of pre-mRNA splicing. RNA.

[27] Malinova A (2017). Assembly of the U5 snRNP component PRPF8 is controlled by the HSP90/R2TP chaperones. J Cell Biol.

[28] Ruzickova S, Stanek D (2017). Mutations in spliceosomal proteins and retina degeneration. RNA Biol.

[29] Castello A (2012). Insights into RNA biology from an atlas of mammalian mRNA-binding proteins. Cell.

[30] Candiano G (2004). Blue silver: a very sensitive colloidal Coomassie G-250 staining for proteome analysis. Electrophoresis.

[31] D’Ambrosio C (2008). A proteomic characterization of water buffalo milk fractions describing PTM of major species and the identification of minor components involved in nutrient delivery and defense against pathogens. Proteomics.

[32] Schmittgen TD, Livak KJ (2008). Analyzing real-time PCR data by the comparative C(T) method. Nature protocols.

[33] Falco G, Stanghellini I, Ko MS (2006). Use of Chuk as an internal standard suitable for quantitative RT-PCR in mouse preimplantation embryos. Reproductive biomedicine online.

