# Supplementary figures and images for: DNAJC17 is localized in nuclear speckles and interacts with splicing machinery components
Source: Sci Rep. 2018 May 17;8:7794. doi: 10.1038/s41598-018-26093-1 (PMC5958099; doi:10.1038/s41598-018-26093-1)

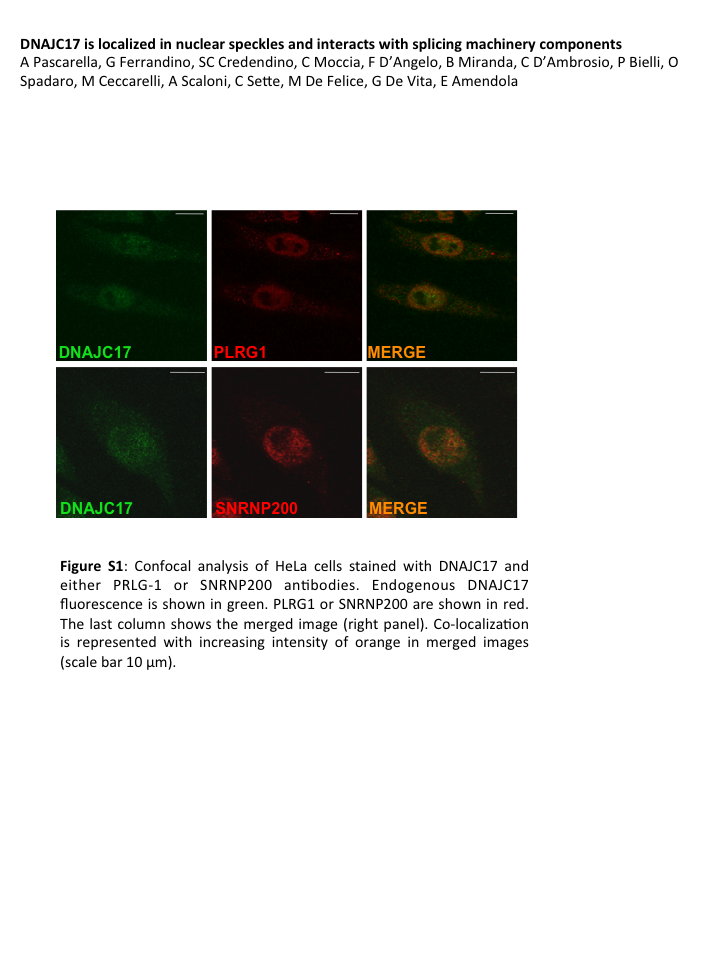

Supplement: Supplementary file 1 — Figure S1 [file 41598_2018_26093_MOESM1_ESM.tif]
